# (2,2′-Bipyridine)bis­(triphenyl­phosphine)copper(I) nitrate chloro­form solvate hemihydrate

**DOI:** 10.1107/S1600536808006260

**Published:** 2008-03-12

**Authors:** Maribel Navarro, Oscar A. Corona, Teresa González, Mario V. Capparelli

**Affiliations:** a Centro de Química, Instituto Venezolano de Investigaciones Científicas, Caracas, Venezuela; b Departamento de Química, Facultad de Ciencias, Universidad Central de Venezuela, Caracas, Venezuela

## Abstract

In the title compound, [Cu(C_10_H_8_N_2_)(C_18_H_15_P)_2_]NO_2_·CHCl_3_·0.5H_2_O, the Cu atom is tetra­hedrally coordinated by a bidentate 2,2′-bipyridine ligand and two PPh_3_ ligands. The Cu—N and Cu—P distances are similar to those observed in similar compounds. The range of coordination angles shows a moderate distortion from ideal tetra­hedral geometry. The bipyridine ligand is twisted [14.2 (4)°] about the ring–ring C—C bond. The nitrate anion and the water and chloro­form mol­ecules of solvation are disordered. In the crystal structure, there are O(water)—H⋯O(nitrate), C—H⋯O(water) and C—H⋯O(nitrate) hydrogen bonds.

## Related literature

For related literature, see: Allen *et al.* (1987 ▶); Engelhardt *et al.* (1985 ▶); Hirshfeld (1976 ▶); Navarro *et al.* (2003 ▶). 
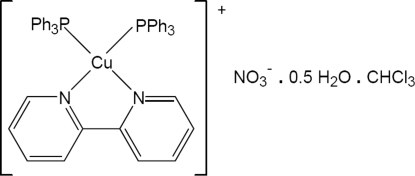


## Experimental

### 

#### Crystal data

[Cu(C_10_H_8_N_2_)(C_18_H_15_P)_2_]NO_3_·CHCl_3_·0.5H_2_O
*M* *_r_* = 934.65
Triclinic, 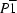

*a* = 10.754 (2) Å
*b* = 12.672 (3) Å
*c* = 17.464 (4) Å
α = 99.100 (5)°
β = 99.279 (4)°
γ = 101.229 (5)°
*V* = 2259.4 (9) Å^3^
*Z* = 2
Mo *K*α radiation
μ = 0.78 mm^−1^
*T* = 296 (2) K
0.56 × 0.51 × 0.40 mm

#### Data collection

Rigaku AFC7S Mercury diffractometer
Absorption correction: multi-scan (*CrystalClear*; Rigaku/MSC, 2005 ▶) *T*_min_ = 0.584, *T*_max_ = 0.733
25842 measured reflections
8576 independent reflections
6719 reflections with *I* > 2σ(*I*)
*R*_int_ = 0.031

#### Refinement

*R*[*F* ^2^ > 2σ(*F* ^2^)] = 0.051
*wR*(*F* ^2^) = 0.136
*S* = 1.06
8576 reflections
549 parameters
20 restraints
H-atom parameters constrained
Δρ_max_ = 0.49 e Å^−3^
Δρ_min_ = −0.53 e Å^−3^

### 

Data collection: *CrystalClear* (Rigaku/MSC, 2005 ▶); cell refinement: *CrystalClear*; data reduction: *CrystalClear*; program(s) used to solve structure: *SHELXS97* (Sheldrick, 2008 ▶); program(s) used to refine structure: *SHELXL97* (Sheldrick, 2008 ▶); molecular graphics: *ORTEP-3* (Farrugia, 1997 ▶); software used to prepare material for publication: *PLATON* (Spek, 2003 ▶) and *WinGX* (Farrugia, 1999 ▶).

## Supplementary Material

Crystal structure: contains datablocks global, I. DOI: 10.1107/S1600536808006260/bg2166sup1.cif

Structure factors: contains datablocks I. DOI: 10.1107/S1600536808006260/bg2166Isup2.hkl

Additional supplementary materials:  crystallographic information; 3D view; checkCIF report

## Figures and Tables

**Table:** 

Cu1—N1	2.070 (2)
Cu1—N12	2.103 (3)
Cu1—P2	2.2600 (9)
Cu1—P1	2.2659 (9)

**Table:** 

N1—Cu1—N12	79.71 (10)
N1—Cu1—P2	111.18 (8)
N12—Cu1—P2	111.86 (7)
N1—Cu1—P1	111.46 (8)
N12—Cu1—P1	108.68 (7)
P2—Cu1—P1	124.89 (3)

**Table:** 

N1—C6—C7—N12	14.2 (4)

**Table 2:** Hydrogen-bond geometry (Å, °)

*D*—H⋯*A*	*D*—H	H⋯*A*	*D*⋯*A*	*D*—H⋯*A*
C2—H2⋯O3*S*^i^	0.93	2.56	3.317 (9)	139
C5—H5⋯O1*W*^i^	0.93	2.34	3.182 (15)	151
C8—H8⋯O2*S*^ii^	0.93	2.38	3.242 (11)	155
C45—H45⋯O3*S*	0.93	2.59	3.493 (10)	163
C1*S*—H1*S*⋯O1*S*^iii^	0.98	2.25	3.204 (7)	165
O1*W*⋯O2*S*^iv^			2.663 (19)	
O1*W*⋯O2*S*^v^			2.667 (19)	

Symmetry codes: (i) 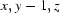; (ii) 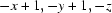; (iii) 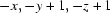; (iv) 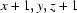; (v) 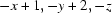.

**Fig. 1.**
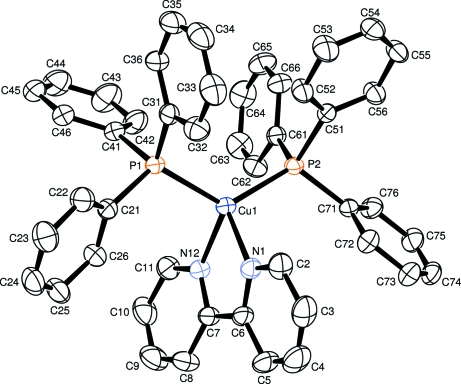
Structure of the complex cation [Cu(PPh3)2(bipy)]+ showing the atomic numbering. Displacement parameters are drawn at 50% probability level.

**Fig. 2.**
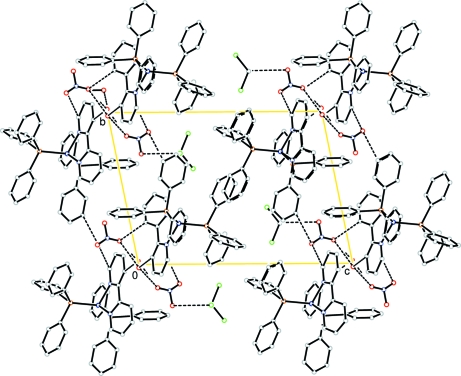
Crystal structure of (I). For clarity, alternative positions for disordered atoms and H atoms were omitted. Possible hydrogen bonds are shown as dashed lines.

## supplementary crystallographic information

### Comment

The title compound (I) was prepared within our program of studies of copper complexes containing N-bidentated aromatic ligands, focused on the search for drugs with biological activity, especially against parasitic diseases (Navarro *et al.*, 2003).

The structure analysis showed that, in addition to the complex cation and the (partially disordered) nitrate anion the crystals contain disordered molecules of water and chloroform of solvation.

In the cation (Fig. 1), the metal atom is tetrahedrally coordinated to a bidentated 2,2'-bipyridine (bipy) ligand and to two PPh_3_ moieties. The Cu—N and Cu—P (Table 1) distances are comparable to Cu—N, 2.056 (8), 2.113 (9) Å and Cu—P, 2.246 (3), 2.256 (3) Å observed in the same cation in [Cu(PPh_3_)_2_(bipy)]ClO_4_ (Engelhardt *et al.*, 1985). All bond lengths and angles in the organic ligands are within normal values (Allen *et al.*, 1987).

The range of coordination angles (Table 1) shows a moderate distortion from the ideal tetrahedral geometry. As expected, the bite angle is the smaller one, while the P—Cu—P angle is the largest, due to the bulkiness of the PPh_3_ moieties. The five-member metallacycle, Cu1—N1—C6—C7—N2, can be described as an envelope on C6, albeit a flat one [C6 is 0.148 (4) Å out of the plane]. The bipy ligand is twisted about C6—C7 (Table 1); the dihedral angle between both heterocycles is 17.0 (2)°.

Although the water's H atoms could not be located (see Refinement Section), short contacts with the nitrate anion [O1w···O2s(1 + *x*, *y*, *z*), 2.663 (19) Å; O1w···O2s(1 - *x*, 2 - *y*, -*z*), 2.667 (19) Å] indicate hydrogen bonds between these two groups. Further evidence of the feasibility of these links is given by the corresponding O2s···O1w···O2s angle [146.7 (5)°]. Several C—H···O bonds may also contribute to the crystal packing (Fig. 2 and Supplementary material).

### Experimental

The title compound (I) was synthesized by the reaction of copper nitrate with bipyridine. To a solution of Cu(PPh_3_)_2_NO_3_ (100 mg, 0.15 mmol) in dichloromethane (10 ml) was added 2,2'-bipyridine (24 mg, 0.15 mmol). The solution, initially transparent, became yellow. It was stirred for 1 h at room temperature and then was added to hexane (50 ml). The light yellow solid formed was filtered and dried (122 mg, 98%). All operations were carried out under inert atmosphere. Crystals suitable for X-ray analysis were obtained by slow evaporation of a chloroform solution.

### Refinement

The hydrogen atoms were placed in calculated positions using a riding atom model with fixed C—H distances [0.93 Å for C(*sp*^2^), 0.98 Å for C(*sp*^3^) in CHCl_3_] and *U*_iso_ = 1.2 *U*_eq_(parent atom).

The nitrate anion and the water and chloroform molecules of solvation were found to be disordered. The NO_3_^-^ showed severe disorder, difficult to model satisfactorily; in the final refinement the four largest residual electron density peaks were close (0.64–1.07 e/Å^3^) to NO_3_ atoms. O2s and O3s were split in two positions, with complementary occupancies, and refined isotropically to final occupancies of 0.505 (14) and 0.719 (12) respectively. To obtain better geometries, restraints were applied: SADI to all N—O bonds and to all O···O distances, and FLAT to both NO_3_ groups. An attempt at splitting N1s, as suggested by its elongated ADP, gave meaningless results. The O1w atom of the water molecule was given an occupancy of 1/2, since it is disordered between two centrosymmetrically related positions, which are mutually exclusive [O1w···O1w(2 - *x*, 2 - *y*, -*x*), 1.53 (3) Å]. The corresponding H atoms could not be found. Each of the Cl atoms of chloroform was split in two alternative positions, with complementary occupancies. The main positions [final occupancy 0.911 (6) for all three] were refined anisotropically, while the alternative ones were given isotropic displacement parameters.

Both Cu—P bonds, with ΔU/σ = 7.21 for Cu1—P1 and 7.13 for Cu1—P2, failed to pass the standard rigid-bond test (*e.g.*: ΔU/σ≤ 5, Spek, 1998; Hirshfeld, 1976) even after applying a DELU restraint (Sheldrick, 2008). This was probably due to an unfavorable specimen morphology which caused a poor absorption correction.

### Crystal data

**Table:**

[Cu(C_10_H_8_N_2_)(C_18_H_15_P)_2_]NO_3_·CHCl_3_·0.5H_2_O	*Z* = 2
*M**_r_* = 934.65	*F*_000_ = 962
Triclinic, *P*1	*D*_x_ = 1.374 Mg m^−^^3^
Hall symbol: -P 1	Mo *K*α radiation λ = 0.71070 Å
*a* = 10.754 (2) Å	Cell parameters from 15269 reflections
*b* = 12.672 (3) Å	θ = 1.7–28.0º
*c* = 17.464 (4) Å	µ = 0.78 mm^−^^1^
α = 99.100 (5)º	*T* = 296 (2) K
β = 99.279 (4)º	Irregular, green
γ = 101.229 (5)º	0.56 × 0.51 × 0.40 mm
*V* = 2259.4 (9) Å^3^	

### Data collection

**Table:**

Rigaku AFC7S Mercury diffractometer	8576 independent reflections
Radiation source: fine-focus sealed tube	6719 reflections with *I* > 2σ(*I*)
Monochromator: graphite	*R*_int_ = 0.031
Detector resolution: 14.63 pixels mm^-1^	θ_max_ = 28.0º
*T* = 296(2) K	θ_min_ = 1.2º
ω scans	*h* = −13→13
Absorption correction: multi-scan(CrystalClear; Rigaku/MSC, 2005)	*k* = −16→15
*T*_min_ = 0.584, *T*_max_ = 0.733	*l* = −21→21
25842 measured reflections	

### Refinement

**Table:**

Refinement on *F*^2^	Secondary atom site location: difference Fourier map
Least-squares matrix: full	Hydrogen site location: inferred from neighbouring sites
*R*[*F*^2^ > 2σ(*F*^2^)] = 0.051	H-atom parameters constrained
*wR*(*F*^2^) = 0.136	*w* = 1/[σ^2^(*F*_o_^2^) + (0.0572*P*)^2^ + 1.3813*P*] where *P* = (*F*_o_^2^ + 2*F*_c_^2^)/3
*S* = 1.06	(Δ/σ)_max_ = 0.001
8576 reflections	Δρ_max_ = 0.49 e Å^−^^3^
549 parameters	Δρ_min_ = −0.52 e Å^−^^3^
20 restraints	Extinction correction: none
Primary atom site location: structure-invariant direct methods	

### Special details

**Table:**

Geometry. All e.s.d.'s (except the e.s.d. in the dihedral angle between two l.s. planes) are estimated using the full covariance matrix. The cell e.s.d.'s are taken into account individually in the estimation of e.s.d.'s in distances, angles and torsion angles; correlations between e.s.d.'s in cell parameters are only used when they are defined by crystal symmetry. An approximate (isotropic) treatment of cell e.s.d.'s is used for estimating e.s.d.'s involving l.s. planes.
Refinement. Refinement of *F*^2^ against ALL reflections. The weighted *R*-factor *wR* and goodness of fit *S* are based on *F*^2^, conventional *R*-factors *R* are based on *F*, with *F* set to zero for negative *F*^2^. The threshold expression of *F*^2^ > σ(*F*^2^) is used only for calculating *R*-factors(gt) *etc*. and is not relevant to the choice of reflections for refinement. *R*-factors based on *F*^2^ are statistically about twice as large as those based on *F*, and *R*- factors based on ALL data will be even larger. Refinement details for disordered atoms are given in the Refimnement section.

### Fractional atomic coordinates and isotropic or equivalent isotropic displacement parameters (Å^2^)

**Table:**

	*x*	*y*	*z*	*U*_iso_*/*U*_eq_	Occ. (<1)
Cu1	0.56159 (3)	0.26669 (3)	0.22887 (2)	0.04336 (12)	
P1	0.40007 (8)	0.34553 (6)	0.18315 (5)	0.04467 (19)	
P2	0.61841 (7)	0.24899 (6)	0.35575 (4)	0.04036 (18)	
N1	0.5630 (3)	0.1260 (2)	0.15089 (15)	0.0517 (6)	
C2	0.4854 (4)	0.0263 (3)	0.1418 (2)	0.0661 (10)	
H2	0.4128	0.0195	0.1648	0.079*	
C3	0.5102 (5)	−0.0673 (3)	0.0989 (3)	0.0839 (13)	
H3	0.4550	−0.1356	0.0934	0.101*	
C4	0.6161 (6)	−0.0569 (4)	0.0654 (3)	0.0951 (15)	
H4	0.6346	−0.1187	0.0372	0.114*	
C5	0.6960 (5)	0.0441 (4)	0.0730 (2)	0.0810 (12)	
H5	0.7687	0.0517	0.0500	0.097*	
C6	0.6661 (3)	0.1351 (3)	0.11567 (19)	0.0555 (8)	
C7	0.7456 (3)	0.2477 (3)	0.12513 (19)	0.0549 (8)	
C8	0.8316 (4)	0.2757 (4)	0.0758 (2)	0.0813 (12)	
H8	0.8441	0.2222	0.0367	0.098*	
C9	0.8968 (4)	0.3815 (5)	0.0854 (3)	0.0913 (14)	
H9	0.9547	0.4007	0.0531	0.110*	
C10	0.8769 (4)	0.4593 (4)	0.1426 (3)	0.0830 (13)	
H10	0.9203	0.5322	0.1495	0.100*	
C11	0.7911 (3)	0.4281 (3)	0.1902 (2)	0.0625 (9)	
H11	0.7791	0.4811	0.2299	0.075*	
N12	0.7239 (2)	0.3238 (2)	0.18134 (15)	0.0496 (6)	
C21	0.3703 (3)	0.3373 (3)	0.07633 (18)	0.0506 (7)	
C22	0.2493 (4)	0.3103 (3)	0.0274 (2)	0.0727 (10)	
H22	0.1753	0.2938	0.0484	0.087*	
C23	0.2388 (5)	0.3081 (4)	−0.0532 (2)	0.0900 (14)	
H23	0.1573	0.2884	−0.0859	0.108*	
C24	0.3442 (5)	0.3339 (3)	−0.0849 (2)	0.0841 (13)	
H24	0.3351	0.3340	−0.1387	0.101*	
C25	0.4636 (5)	0.3597 (3)	−0.0381 (2)	0.0769 (11)	
H25	0.5364	0.3766	−0.0601	0.092*	
C26	0.4774 (4)	0.3610 (3)	0.0419 (2)	0.0605 (9)	
H26	0.5599	0.3780	0.0733	0.073*	
C31	0.2417 (3)	0.2894 (3)	0.20327 (19)	0.0506 (7)	
C32	0.1871 (3)	0.1791 (3)	0.1751 (2)	0.0676 (10)	
H32	0.2279	0.1370	0.1431	0.081*	
C33	0.0709 (4)	0.1305 (4)	0.1943 (3)	0.0825 (12)	
H33	0.0345	0.0563	0.1753	0.099*	
C34	0.0109 (4)	0.1929 (5)	0.2412 (3)	0.0854 (14)	
H34	−0.0662	0.1608	0.2544	0.103*	
C35	0.0637 (4)	0.3019 (4)	0.2687 (3)	0.0831 (13)	
H35	0.0221	0.3438	0.3001	0.100*	
C36	0.1787 (3)	0.3507 (3)	0.2502 (2)	0.0633 (9)	
H36	0.2140	0.4251	0.2694	0.076*	
C41	0.4297 (3)	0.4929 (2)	0.22167 (19)	0.0509 (7)	
C42	0.5256 (4)	0.5401 (3)	0.2877 (2)	0.0698 (10)	
H42	0.5735	0.4964	0.3119	0.084*	
C43	0.5515 (5)	0.6512 (4)	0.3184 (3)	0.0949 (15)	
H43	0.6155	0.6819	0.3633	0.114*	
C44	0.4819 (5)	0.7157 (4)	0.2822 (3)	0.0934 (14)	
H44	0.5004	0.7907	0.3022	0.112*	
C45	0.3853 (5)	0.6713 (3)	0.2168 (3)	0.0797 (12)	
H45	0.3376	0.7158	0.1934	0.096*	
C46	0.3594 (4)	0.5600 (3)	0.1862 (2)	0.0618 (9)	
H46	0.2947	0.5298	0.1417	0.074*	
C51	0.4918 (3)	0.1884 (2)	0.40385 (17)	0.0422 (6)	
C52	0.3641 (3)	0.1788 (3)	0.3692 (2)	0.0585 (9)	
H52	0.3439	0.2004	0.3211	0.070*	
C53	0.2666 (4)	0.1371 (4)	0.4059 (3)	0.0776 (12)	
H53	0.1810	0.1313	0.3824	0.093*	
C54	0.2936 (4)	0.1043 (3)	0.4756 (2)	0.0666 (10)	
H54	0.2268	0.0769	0.4999	0.080*	
C55	0.4182 (4)	0.1115 (3)	0.5100 (2)	0.0630 (9)	
H55	0.4366	0.0882	0.5576	0.076*	
C56	0.5182 (3)	0.1533 (3)	0.4745 (2)	0.0583 (8)	
H56	0.6034	0.1578	0.4983	0.070*	
C61	0.6926 (3)	0.3802 (2)	0.42251 (17)	0.0446 (7)	
C62	0.8032 (3)	0.4444 (3)	0.4071 (2)	0.0597 (9)	
H62	0.8394	0.4181	0.3652	0.072*	
C63	0.8595 (4)	0.5465 (3)	0.4535 (3)	0.0743 (11)	
H63	0.9344	0.5877	0.4434	0.089*	
C64	0.8059 (4)	0.5877 (3)	0.5145 (3)	0.0793 (12)	
H64	0.8435	0.6570	0.5453	0.095*	
C65	0.6963 (4)	0.5260 (3)	0.5297 (2)	0.0742 (11)	
H65	0.6595	0.5539	0.5708	0.089*	
C66	0.6399 (3)	0.4228 (3)	0.4846 (2)	0.0575 (8)	
H66	0.5660	0.3817	0.4960	0.069*	
C71	0.7379 (3)	0.1653 (2)	0.37355 (18)	0.0444 (7)	
C72	0.7141 (3)	0.0629 (3)	0.3242 (2)	0.0583 (8)	
H72	0.6405	0.0402	0.2843	0.070*	
C73	0.7991 (4)	−0.0051 (3)	0.3339 (2)	0.0678 (10)	
H73	0.7824	−0.0733	0.3005	0.081*	
C74	0.9088 (3)	0.0279 (3)	0.3931 (2)	0.0657 (10)	
H74	0.9666	−0.0173	0.3993	0.079*	
C75	0.9312 (3)	0.1275 (3)	0.4421 (2)	0.0645 (9)	
H75	1.0039	0.1494	0.4827	0.077*	
C76	0.8469 (3)	0.1965 (3)	0.4323 (2)	0.0558 (8)	
H76	0.8643	0.2646	0.4659	0.067*	
C1S	−0.0946 (4)	0.2653 (3)	0.6980 (3)	0.0822 (12)	
H1S	−0.1093	0.2748	0.7524	0.099*	
Cl1S	0.07297 (19)	0.27094 (17)	0.69940 (16)	0.1137 (7)	0.911 (6)
Cl2S	−0.18878 (19)	0.13990 (18)	0.64402 (13)	0.1167 (7)	0.911 (6)
Cl3S	−0.1396 (2)	0.37078 (17)	0.65417 (17)	0.1141 (9)	0.911 (6)
Cl1T	0.0416 (19)	0.2325 (18)	0.7246 (11)	0.094 (6)*	0.089 (6)
Cl2T	−0.162 (2)	0.191 (3)	0.6233 (15)	0.130 (8)*	0.089 (6)
Cl3T	−0.0968 (14)	0.3998 (12)	0.7051 (13)	0.083 (5)*	0.089 (6)
N1S	0.1383 (10)	0.8269 (6)	0.1199 (3)	0.157 (3)	
O1S	0.1002 (5)	0.7275 (5)	0.1184 (3)	0.1440 (16)	
O2S	0.0559 (12)	0.8453 (8)	0.0668 (6)	0.146 (5)*	0.505 (14)
O2S'	0.1575 (9)	0.9082 (8)	0.0846 (5)	0.123 (4)*	0.495 (14)
O3S	0.2184 (9)	0.8712 (7)	0.1718 (6)	0.182 (4)*	0.719 (12)
O3S'	0.0415 (18)	0.8635 (15)	0.1616 (12)	0.162 (9)*	0.281 (12)
O1W	0.9283 (12)	0.9778 (13)	−0.0008 (10)	0.240 (6)*	0.50

### Atomic displacement parameters (Å^2^)

**Table:**

	*U*^11^	*U*^22^	*U*^33^	*U*^12^	*U*^13^	*U*^23^
Cu1	0.0457 (2)	0.0458 (2)	0.0419 (2)	0.01544 (16)	0.01346 (16)	0.00713 (15)
P1	0.0477 (4)	0.0513 (4)	0.0405 (4)	0.0202 (3)	0.0129 (3)	0.0096 (3)
P2	0.0363 (4)	0.0465 (4)	0.0409 (4)	0.0123 (3)	0.0109 (3)	0.0093 (3)
N1	0.0652 (17)	0.0463 (14)	0.0434 (15)	0.0186 (13)	0.0071 (13)	0.0042 (11)
C2	0.080 (2)	0.052 (2)	0.059 (2)	0.0102 (18)	0.0028 (19)	0.0029 (16)
C3	0.116 (4)	0.049 (2)	0.074 (3)	0.016 (2)	−0.004 (3)	0.0004 (18)
C4	0.136 (4)	0.068 (3)	0.084 (3)	0.048 (3)	0.018 (3)	−0.006 (2)
C5	0.109 (3)	0.081 (3)	0.065 (3)	0.052 (3)	0.027 (2)	0.003 (2)
C6	0.070 (2)	0.062 (2)	0.0399 (18)	0.0328 (17)	0.0117 (16)	0.0060 (14)
C7	0.0579 (19)	0.070 (2)	0.0459 (19)	0.0287 (17)	0.0176 (15)	0.0137 (15)
C8	0.089 (3)	0.107 (3)	0.067 (3)	0.040 (3)	0.042 (2)	0.022 (2)
C9	0.082 (3)	0.117 (4)	0.090 (3)	0.019 (3)	0.045 (3)	0.041 (3)
C10	0.073 (3)	0.085 (3)	0.097 (3)	0.005 (2)	0.026 (2)	0.044 (3)
C11	0.059 (2)	0.059 (2)	0.069 (2)	0.0074 (17)	0.0163 (18)	0.0153 (17)
N12	0.0491 (14)	0.0550 (15)	0.0500 (16)	0.0185 (12)	0.0147 (12)	0.0125 (12)
C21	0.062 (2)	0.0529 (17)	0.0414 (18)	0.0228 (15)	0.0111 (15)	0.0106 (13)
C22	0.068 (2)	0.101 (3)	0.057 (2)	0.035 (2)	0.0095 (19)	0.023 (2)
C23	0.095 (3)	0.121 (4)	0.052 (3)	0.034 (3)	−0.010 (2)	0.021 (2)
C24	0.128 (4)	0.083 (3)	0.049 (2)	0.035 (3)	0.015 (3)	0.022 (2)
C25	0.105 (3)	0.077 (3)	0.059 (2)	0.023 (2)	0.031 (2)	0.027 (2)
C26	0.071 (2)	0.067 (2)	0.049 (2)	0.0191 (18)	0.0166 (17)	0.0155 (16)
C31	0.0467 (17)	0.063 (2)	0.0479 (19)	0.0194 (15)	0.0093 (14)	0.0190 (15)
C32	0.058 (2)	0.078 (3)	0.066 (2)	0.0139 (19)	0.0105 (18)	0.0127 (19)
C33	0.063 (2)	0.086 (3)	0.091 (3)	−0.003 (2)	0.005 (2)	0.031 (2)
C34	0.053 (2)	0.132 (4)	0.084 (3)	0.020 (3)	0.021 (2)	0.051 (3)
C35	0.071 (3)	0.123 (4)	0.080 (3)	0.044 (3)	0.037 (2)	0.042 (3)
C36	0.061 (2)	0.080 (2)	0.063 (2)	0.0306 (19)	0.0269 (18)	0.0231 (18)
C41	0.0577 (19)	0.0502 (17)	0.0527 (19)	0.0214 (15)	0.0208 (15)	0.0117 (14)
C42	0.079 (3)	0.060 (2)	0.066 (2)	0.0253 (19)	0.006 (2)	−0.0021 (18)
C43	0.106 (4)	0.073 (3)	0.090 (3)	0.025 (3)	0.001 (3)	−0.015 (2)
C44	0.124 (4)	0.058 (2)	0.098 (4)	0.025 (3)	0.036 (3)	−0.002 (2)
C45	0.110 (3)	0.066 (2)	0.091 (3)	0.045 (2)	0.053 (3)	0.031 (2)
C46	0.072 (2)	0.063 (2)	0.063 (2)	0.0302 (18)	0.0256 (18)	0.0188 (17)
C51	0.0431 (15)	0.0408 (15)	0.0455 (17)	0.0129 (12)	0.0112 (13)	0.0100 (12)
C52	0.0426 (17)	0.075 (2)	0.063 (2)	0.0106 (16)	0.0104 (15)	0.0297 (18)
C53	0.0441 (19)	0.102 (3)	0.095 (3)	0.010 (2)	0.022 (2)	0.043 (3)
C54	0.061 (2)	0.067 (2)	0.081 (3)	0.0106 (18)	0.035 (2)	0.0257 (19)
C55	0.077 (3)	0.066 (2)	0.054 (2)	0.0147 (19)	0.0265 (19)	0.0225 (17)
C56	0.0505 (18)	0.075 (2)	0.053 (2)	0.0146 (17)	0.0109 (15)	0.0230 (17)
C61	0.0445 (16)	0.0490 (16)	0.0411 (16)	0.0137 (13)	0.0068 (13)	0.0091 (13)
C62	0.0518 (19)	0.064 (2)	0.059 (2)	0.0031 (16)	0.0134 (16)	0.0092 (16)
C63	0.065 (2)	0.064 (2)	0.081 (3)	−0.0072 (19)	0.009 (2)	0.009 (2)
C64	0.083 (3)	0.056 (2)	0.082 (3)	0.003 (2)	0.001 (2)	−0.003 (2)
C65	0.081 (3)	0.065 (2)	0.071 (3)	0.022 (2)	0.016 (2)	−0.0115 (19)
C66	0.0544 (19)	0.0541 (18)	0.063 (2)	0.0131 (15)	0.0170 (16)	0.0014 (15)
C71	0.0399 (15)	0.0529 (17)	0.0478 (18)	0.0174 (13)	0.0156 (13)	0.0164 (13)
C72	0.0558 (19)	0.0559 (19)	0.065 (2)	0.0204 (16)	0.0077 (17)	0.0125 (16)
C73	0.079 (3)	0.056 (2)	0.078 (3)	0.0294 (19)	0.022 (2)	0.0162 (18)
C74	0.055 (2)	0.071 (2)	0.093 (3)	0.0326 (18)	0.030 (2)	0.041 (2)
C75	0.0427 (18)	0.080 (3)	0.079 (3)	0.0223 (17)	0.0105 (17)	0.030 (2)
C76	0.0440 (17)	0.064 (2)	0.061 (2)	0.0180 (15)	0.0082 (15)	0.0113 (16)
C1S	0.106 (3)	0.072 (3)	0.070 (3)	0.032 (2)	0.015 (2)	0.008 (2)
Cl1S	0.1039 (12)	0.0881 (11)	0.1527 (17)	0.0357 (9)	0.0224 (11)	0.0201 (11)
Cl2S	0.1263 (13)	0.0855 (12)	0.1194 (14)	0.0151 (10)	0.0038 (10)	−0.0035 (10)
Cl3S	0.1557 (17)	0.1091 (12)	0.123 (2)	0.0812 (12)	0.0695 (16)	0.0501 (12)
N1S	0.281 (9)	0.163 (6)	0.063 (3)	0.120 (6)	0.025 (5)	0.049 (4)
O1S	0.138 (4)	0.184 (5)	0.110 (3)	0.059 (4)	0.013 (3)	0.012 (3)

### Geometric parameters (Å, °)

**Table:**

Cu1—N1	2.070 (2)	C42—C43	1.381 (5)
Cu1—N12	2.103 (3)	C42—H42	0.9300
Cu1—P2	2.2600 (9)	C43—C44	1.370 (7)
Cu1—P1	2.2659 (9)	C43—H43	0.9300
P1—C21	1.824 (3)	C44—C45	1.373 (6)
P1—C31	1.825 (3)	C44—H44	0.9300
P1—C41	1.828 (3)	C45—C46	1.383 (5)
P2—C51	1.821 (3)	C45—H45	0.9300
P2—C61	1.825 (3)	C46—H46	0.9300
P2—C71	1.839 (3)	C51—C52	1.382 (4)
N1—C2	1.341 (4)	C51—C56	1.382 (4)
N1—C6	1.347 (4)	C52—C53	1.377 (5)
C2—C3	1.395 (5)	C52—H52	0.9300
C2—H2	0.9300	C53—C54	1.353 (5)
C3—C4	1.356 (7)	C53—H53	0.9300
C3—H3	0.9300	C54—C55	1.357 (5)
C4—C5	1.370 (6)	C54—H54	0.9300
C4—H4	0.9300	C55—C56	1.385 (5)
C5—C6	1.393 (5)	C55—H55	0.9300
C5—H5	0.9300	C56—H56	0.9300
C6—C7	1.483 (5)	C61—C66	1.386 (4)
C7—N12	1.345 (4)	C61—C62	1.394 (4)
C7—C8	1.396 (5)	C62—C63	1.379 (5)
C8—C9	1.357 (6)	C62—H62	0.9300
C8—H8	0.9300	C63—C64	1.371 (6)
C9—C10	1.362 (7)	C63—H63	0.9300
C9—H9	0.9300	C64—C65	1.370 (6)
C10—C11	1.382 (5)	C64—H64	0.9300
C10—H10	0.9300	C65—C66	1.382 (5)
C11—N12	1.349 (4)	C65—H65	0.9300
C11—H11	0.9300	C66—H66	0.9300
C21—C22	1.384 (5)	C71—C76	1.373 (4)
C21—C26	1.388 (5)	C71—C72	1.392 (4)
C22—C23	1.389 (5)	C72—C73	1.382 (5)
C22—H22	0.9300	C72—H72	0.9300
C23—C24	1.347 (6)	C73—C74	1.383 (5)
C23—H23	0.9300	C73—H73	0.9300
C24—C25	1.355 (6)	C74—C75	1.363 (5)
C24—H24	0.9300	C74—H74	0.9300
C25—C26	1.378 (5)	C75—C76	1.386 (5)
C25—H25	0.9300	C75—H75	0.9300
C26—H26	0.9300	C76—H76	0.9300
C31—C32	1.382 (5)	C1S—H1S	0.9800
C31—C36	1.384 (5)	C1S—Cl1S	1.786 (5)
C32—C33	1.398 (5)	C1S—Cl2S	1.741 (4)
C32—H32	0.9300	C1S—Cl3S	1.750 (4)
C33—C34	1.371 (6)	C1S—Cl1t	1.617 (19)
C33—H33	0.9300	C1S—Cl2t	1.47 (3)
C34—C35	1.363 (6)	C1S—Cl3t	1.694 (15)
C34—H34	0.9300	N1S—O3S	1.131 (10)
C35—C36	1.382 (5)	N1S—O1S	1.241 (8)
C35—H35	0.9300	N1S—O2S	1.259 (12)
C36—H36	0.9300	N1S—O2S'	1.282 (10)
C41—C42	1.382 (5)	N1S—O3S'	1.47 (2)
C41—C46	1.394 (5)		
			
N1—Cu1—N12	79.71 (10)	C42—C41—C46	118.5 (3)
N1—Cu1—P2	111.18 (8)	C42—C41—P1	119.2 (3)
N12—Cu1—P2	111.86 (7)	C46—C41—P1	122.2 (3)
N1—Cu1—P1	111.46 (8)	C43—C42—C41	121.0 (4)
N12—Cu1—P1	108.68 (7)	C43—C42—H42	119.5
P2—Cu1—P1	124.89 (3)	C41—C42—H42	119.5
C21—P1—C31	104.14 (15)	C44—C43—C42	119.4 (4)
C21—P1—C41	102.77 (14)	C44—C43—H43	120.3
C31—P1—C41	104.43 (15)	C42—C43—H43	120.3
C21—P1—Cu1	114.07 (11)	C43—C44—C45	121.1 (4)
C31—P1—Cu1	115.68 (10)	C43—C44—H44	119.5
C41—P1—Cu1	114.29 (11)	C45—C44—H44	119.5
C51—P2—C61	103.12 (13)	C44—C45—C46	119.4 (4)
C51—P2—C71	101.93 (13)	C44—C45—H45	120.3
C61—P2—C71	103.69 (14)	C46—C45—H45	120.3
C51—P2—Cu1	118.06 (10)	C45—C46—C41	120.5 (4)
C61—P2—Cu1	112.74 (10)	C45—C46—H46	119.7
C71—P2—Cu1	115.48 (10)	C41—C46—H46	119.7
C2—N1—C6	118.3 (3)	C52—C51—C56	118.5 (3)
C2—N1—Cu1	127.0 (2)	C52—C51—P2	118.9 (2)
C6—N1—Cu1	113.9 (2)	C56—C51—P2	122.6 (2)
N1—C2—C3	122.1 (4)	C53—C52—C51	120.0 (3)
N1—C2—H2	118.9	C53—C52—H52	120.0
C3—C2—H2	118.9	C51—C52—H52	120.0
C4—C3—C2	118.7 (4)	C54—C53—C52	121.0 (4)
C4—C3—H3	120.6	C54—C53—H53	119.5
C2—C3—H3	120.6	C52—C53—H53	119.5
C3—C4—C5	120.3 (4)	C53—C54—C55	119.9 (3)
C3—C4—H4	119.8	C53—C54—H54	120.0
C5—C4—H4	119.8	C55—C54—H54	120.0
C4—C5—C6	118.7 (4)	C54—C55—C56	120.3 (3)
C4—C5—H5	120.7	C54—C55—H55	119.8
C6—C5—H5	120.7	C56—C55—H55	119.8
N1—C6—C5	121.8 (3)	C51—C56—C55	120.2 (3)
N1—C6—C7	116.0 (3)	C51—C56—H56	119.9
C5—C6—C7	122.2 (3)	C55—C56—H56	119.9
N12—C7—C8	121.4 (3)	C66—C61—C62	118.1 (3)
N12—C7—C6	115.8 (3)	C66—C61—P2	123.4 (2)
C8—C7—C6	122.7 (3)	C62—C61—P2	118.4 (2)
C9—C8—C7	119.6 (4)	C63—C62—C61	120.7 (3)
C9—C8—H8	120.2	C63—C62—H62	119.7
C7—C8—H8	120.2	C61—C62—H62	119.7
C8—C9—C10	119.7 (4)	C64—C63—C62	120.5 (4)
C8—C9—H9	120.1	C64—C63—H63	119.7
C10—C9—H9	120.1	C62—C63—H63	119.7
C9—C10—C11	118.8 (4)	C65—C64—C63	119.4 (3)
C9—C10—H10	120.6	C65—C64—H64	120.3
C11—C10—H10	120.6	C63—C64—H64	120.3
N12—C11—C10	122.7 (4)	C64—C65—C66	120.8 (4)
N12—C11—H11	118.6	C64—C65—H65	119.6
C10—C11—H11	118.6	C66—C65—H65	119.6
C7—N12—C11	117.7 (3)	C65—C66—C61	120.5 (3)
C7—N12—Cu1	113.2 (2)	C65—C66—H66	119.8
C11—N12—Cu1	128.0 (2)	C61—C66—H66	119.8
C22—C21—C26	117.8 (3)	C76—C71—C72	118.5 (3)
C22—C21—P1	125.0 (3)	C76—C71—P2	124.5 (2)
C26—C21—P1	117.2 (3)	C72—C71—P2	117.0 (2)
C21—C22—C23	119.7 (4)	C73—C72—C71	120.5 (3)
C21—C22—H22	120.1	C73—C72—H72	119.7
C23—C22—H22	120.1	C71—C72—H72	119.7
C24—C23—C22	121.3 (4)	C72—C73—C74	120.2 (4)
C24—C23—H23	119.3	C72—C73—H73	119.9
C22—C23—H23	119.3	C74—C73—H73	119.9
C23—C24—C25	119.8 (4)	C75—C74—C73	119.3 (3)
C23—C24—H24	120.1	C75—C74—H74	120.3
C25—C24—H24	120.1	C73—C74—H74	120.3
C24—C25—C26	120.3 (4)	C74—C75—C76	120.8 (3)
C24—C25—H25	119.9	C74—C75—H75	119.6
C26—C25—H25	119.9	C76—C75—H75	119.6
C25—C26—C21	121.0 (4)	C71—C76—C75	120.7 (3)
C25—C26—H26	119.5	C71—C76—H76	119.7
C21—C26—H26	119.5	C75—C76—H76	119.7
C32—C31—C36	118.8 (3)	Cl2S—C1S—Cl1S	110.6 (2)
C32—C31—P1	118.3 (3)	Cl2S—C1S—Cl3S	109.0 (3)
C36—C31—P1	122.7 (3)	Cl3S—C1S—Cl1S	109.3 (3)
C31—C32—C33	120.4 (4)	Cl1S—C1S—H1S	109.3
C31—C32—H32	119.8	Cl2S—C1S—H1S	109.3
C33—C32—H32	119.8	Cl3S—C1S—H1S	109.3
C34—C33—C32	119.6 (4)	Cl2t—C1S—Cl1t	107.6 (12)
C34—C33—H33	120.2	Cl2t—C1S—Cl3t	117.1 (11)
C32—C33—H33	120.2	Cl3t—C1S—Cl1t	118.8 (9)
C35—C34—C33	120.2 (4)	Cl1t—C1S—H1S	91.7
C35—C34—H34	119.9	Cl2t—C1S—H1S	134.5
C33—C34—H34	119.9	Cl3t—C1S—H1S	85.6
C34—C35—C36	120.6 (4)	O3S—N1S—O1S	114.4 (7)
C34—C35—H35	119.7	O3S—N1S—O2S	141.2 (10)
C36—C35—H35	119.7	O1S—N1S—O2S	103.1 (9)
C35—C36—C31	120.4 (4)	O1S—N1S—O2S'	151.1 (7)
C35—C36—H36	119.8	O1S—N1S—O3S'	96.4 (10)
C31—C36—H36	119.8	O2S'—N1S—O3S'	96.0 (9)
			
N1—Cu1—P1—C21	−37.02 (14)	C21—P1—C31—C32	67.5 (3)
N12—Cu1—P1—C21	48.98 (14)	C41—P1—C31—C32	175.0 (3)
P2—Cu1—P1—C21	−175.39 (11)	Cu1—P1—C31—C32	−58.5 (3)
N1—Cu1—P1—C31	83.75 (15)	C21—P1—C31—C36	−117.3 (3)
N12—Cu1—P1—C31	169.75 (14)	C41—P1—C31—C36	−9.8 (3)
P2—Cu1—P1—C31	−54.62 (13)	Cu1—P1—C31—C36	116.7 (3)
N1—Cu1—P1—C41	−154.89 (14)	C36—C31—C32—C33	−0.5 (5)
N12—Cu1—P1—C41	−68.88 (14)	P1—C31—C32—C33	174.9 (3)
P2—Cu1—P1—C41	66.75 (12)	C31—C32—C33—C34	0.1 (6)
N1—Cu1—P2—C51	−87.33 (13)	C32—C33—C34—C35	0.4 (6)
N12—Cu1—P2—C51	−174.41 (13)	C33—C34—C35—C36	−0.5 (7)
P1—Cu1—P2—C51	51.13 (11)	C34—C35—C36—C31	0.2 (6)
N1—Cu1—P2—C61	152.51 (14)	C32—C31—C36—C35	0.3 (5)
N12—Cu1—P2—C61	65.43 (13)	P1—C31—C36—C35	−174.8 (3)
P1—Cu1—P2—C61	−69.03 (11)	C21—P1—C41—C42	−140.2 (3)
N1—Cu1—P2—C71	33.56 (14)	C31—P1—C41—C42	111.3 (3)
N12—Cu1—P2—C71	−53.51 (13)	Cu1—P1—C41—C42	−16.1 (3)
P1—Cu1—P2—C71	172.03 (11)	C21—P1—C41—C46	39.2 (3)
N12—Cu1—N1—C2	174.9 (3)	C31—P1—C41—C46	−69.3 (3)
P2—Cu1—N1—C2	65.3 (3)	Cu1—P1—C41—C46	163.3 (2)
P1—Cu1—N1—C2	−79.0 (3)	C46—C41—C42—C43	0.2 (6)
N12—Cu1—N1—C6	5.6 (2)	P1—C41—C42—C43	179.6 (3)
P2—Cu1—N1—C6	−104.0 (2)	C41—C42—C43—C44	−0.8 (7)
P1—Cu1—N1—C6	111.7 (2)	C42—C43—C44—C45	1.3 (8)
C6—N1—C2—C3	1.6 (5)	C43—C44—C45—C46	−1.2 (7)
Cu1—N1—C2—C3	−167.3 (3)	C44—C45—C46—C41	0.6 (6)
N1—C2—C3—C4	0.0 (6)	C42—C41—C46—C45	−0.2 (5)
C2—C3—C4—C5	−0.8 (7)	P1—C41—C46—C45	−179.6 (3)
C3—C4—C5—C6	0.2 (7)	C61—P2—C51—C52	110.3 (3)
C2—N1—C6—C5	−2.3 (5)	C71—P2—C51—C52	−142.3 (3)
Cu1—N1—C6—C5	168.0 (3)	Cu1—P2—C51—C52	−14.7 (3)
C2—N1—C6—C7	177.7 (3)	C61—P2—C51—C56	−68.6 (3)
Cu1—N1—C6—C7	−12.0 (3)	C71—P2—C51—C56	38.8 (3)
C4—C5—C6—N1	1.4 (6)	Cu1—P2—C51—C56	166.4 (2)
C4—C5—C6—C7	−178.5 (4)	C56—C51—C52—C53	1.4 (5)
N1—C6—C7—N12	14.2 (4)	P2—C51—C52—C53	−177.6 (3)
C5—C6—C7—N12	−165.8 (3)	C51—C52—C53—C54	−0.5 (6)
N1—C6—C7—C8	−161.7 (3)	C52—C53—C54—C55	−0.6 (6)
C5—C6—C7—C8	18.3 (5)	C53—C54—C55—C56	0.7 (6)
N12—C7—C8—C9	1.2 (6)	C52—C51—C56—C55	−1.2 (5)
C6—C7—C8—C9	176.8 (4)	P2—C51—C56—C55	177.7 (3)
C7—C8—C9—C10	−0.5 (7)	C54—C55—C56—C51	0.2 (6)
C8—C9—C10—C11	0.6 (7)	C51—P2—C61—C66	−10.6 (3)
C9—C10—C11—N12	−1.4 (6)	C71—P2—C61—C66	−116.6 (3)
C8—C7—N12—C11	−1.9 (5)	Cu1—P2—C61—C66	117.8 (3)
C6—C7—N12—C11	−177.9 (3)	C51—P2—C61—C62	173.9 (3)
C8—C7—N12—Cu1	167.0 (3)	C71—P2—C61—C62	67.9 (3)
C6—C7—N12—Cu1	−9.0 (4)	Cu1—P2—C61—C62	−57.7 (3)
C10—C11—N12—C7	2.0 (5)	C66—C61—C62—C63	1.2 (5)
C10—C11—N12—Cu1	−164.9 (3)	P2—C61—C62—C63	176.9 (3)
N1—Cu1—N12—C7	2.2 (2)	C61—C62—C63—C64	−1.5 (6)
P2—Cu1—N12—C7	111.0 (2)	C62—C63—C64—C65	0.8 (7)
P1—Cu1—N12—C7	−107.2 (2)	C63—C64—C65—C66	0.3 (7)
N1—Cu1—N12—C11	169.6 (3)	C64—C65—C66—C61	−0.6 (6)
P2—Cu1—N12—C11	−81.5 (3)	C62—C61—C66—C65	−0.1 (5)
P1—Cu1—N12—C11	60.3 (3)	P2—C61—C66—C65	−175.6 (3)
C31—P1—C21—C22	9.1 (3)	C51—P2—C71—C76	−99.3 (3)
C41—P1—C21—C22	−99.6 (3)	C61—P2—C71—C76	7.6 (3)
Cu1—P1—C21—C22	136.1 (3)	Cu1—P2—C71—C76	131.4 (2)
C31—P1—C21—C26	−171.6 (3)	C51—P2—C71—C72	79.9 (3)
C41—P1—C21—C26	79.7 (3)	C61—P2—C71—C72	−173.2 (2)
Cu1—P1—C21—C26	−44.6 (3)	Cu1—P2—C71—C72	−49.4 (3)
C26—C21—C22—C23	−0.1 (6)	C76—C71—C72—C73	−0.5 (5)
P1—C21—C22—C23	179.1 (3)	P2—C71—C72—C73	−179.8 (3)
C21—C22—C23—C24	−1.4 (7)	C71—C72—C73—C74	0.1 (5)
C22—C23—C24—C25	1.9 (7)	C72—C73—C74—C75	0.8 (6)
C23—C24—C25—C26	−0.8 (7)	C73—C74—C75—C76	−1.3 (5)
C24—C25—C26—C21	−0.7 (6)	C72—C71—C76—C75	0.0 (5)
C22—C21—C26—C25	1.1 (5)	P2—C71—C76—C75	179.2 (3)
P1—C21—C26—C25	−178.2 (3)	C74—C75—C76—C71	0.9 (5)

### Hydrogen-bond geometry (Å, °)

**Table:**

*D*—H···*A*	*D*—H	H···*A*	*D*···*A*	*D*—H···*A*
C2—H2···O3S^i^	0.93	2.56	3.317 (9)	139
C5—H5···O1W^i^	0.93	2.34	3.182 (15)	151
C8—H8···O2S^ii^	0.93	2.38	3.242 (11)	155
C45—H45···O3S	0.93	2.59	3.493 (10)	163
C1S—H1S···O1S^iii^	0.98	2.25	3.204 (7)	165
O1W—?···O2S^iv^	?	?	2.663 (19)	?
O1W—?···O2S^v^	?	?	2.667 (19)	?

Symmetry codes: (i) *x*, *y*−1, *z*; (ii) −*x*+1, −*y*+1, −*z*; (iii) −*x*, −*y*+1, −*z*+1; (iv) *x*+1, *y*, *z*+1; (v) −*x*+1, −*y*+2, −*z*.
